# Prognostic impact of matched preoperative plasma and serum VEGF in patients with primary colorectal carcinoma

**DOI:** 10.1038/sj.bjc.6600075

**Published:** 2002-02-01

**Authors:** K Werther, I J Christensen, H J Nielsen, and the Danish RANX05 Colorectal Cancer Study Group

**Affiliations:** Department of Surgical Gastroenterology 435, Hvidovre University Hospital, University of Copenhagen, 2650 Hvidovre, Denmark; The Finsen Laboratory, Rigshospitalet, 49 Strandboulevarden, DK-2100 Copenhagen, Denmark

**Keywords:** angiogenesis, colorectal cancer, prognosis, VEGF

## Abstract

In serum, the major part of vascular endothelial growth factor derives from *in vitro* degranulation of granulocytes and platelets. Therefore, plasma may be preferred for vascular endothelial growth factor measurements. However, which specimen is the best predictor of survival is still debated. The present study analyzed the prognostic value of matched preoperative serum and plasma vascular endothelial growth factor concentrations in patients with colorectal cancer. To establish the reference range among healthy people, vascular endothelial growth factor was analyzed in 50 matched EDTA-plasma and serum samples from healthy blood donors. Preoperatively, in 524 patients with colorectal cancer, matched plasma and serum vascular endothelial growth factor concentrations were analyzed. In the colorectal cancer patients, the median plasma vascular endothelial growth factor concentration (44 pg ml^−1^) was significantly (*P*=0.01) higher than the median plasma vascular endothelial growth factor concentration (30 pg ml^−1^) in the healthy blood donors. In serum, no significant (*P*=0.30) difference in the median vascular endothelial growth factor concentration was found between colorectal cancer patients (268 pg ml^−1^) and healthy blood donors (220 pg ml^−1^). The preoperative vascular endothelial growth factor concentrations were dichotomized by the 95th percentile of the healthy blood donors (plasma=112 pg ml^−1^, serum=533 pg ml^−1^). In univariate survival analyses, both high plasma vascular endothelial growth factor (>112 pg ml^−1^) and high serum vascular endothelial growth factor (>533 pg ml^−1^) predicted a reduced survival. In multivariate survival analyses, high serum vascular endothelial growth factor (>533 pg ml^−1^) independently predicted a reduced survival (HR=1.65, *P*=0.015), while high plasma vascular endothelial growth factor (>112 pg ml^−1^) did not (HR=1.27, *P*=0.23). This study indicates that preoperative serum vascular endothelial growth factor apparently is a better predictor of overall survival than the preoperative plasma vascular endothelial growth factor.

*British Journal of Cancer* (2002) **86**, 417–423. DOI: 10.1038/sj/bjc/6600075 www.bjcancer.com

© 2002 The Cancer Research Campaign

## 

Vascular endothelial growth factor (VEGF) is one of the strongest promoters of angiogenesis, and it has been indicated, that the preoperative serum VEGF concentration is a prognostic marker in a variety of solid tumours (Salven *et al*, 1999a; Chin *et al*, 2000). In a previous study including 614 patients, it was shown that preoperative serum VEGF concentration, independent of Dukes stage, was a strong predictor of overall survival of patients with colorectal cancer (CRC) (Werther *et al*, 2000). However, VEGF is stored in circulating white blood cells and platelets (Nielsen *et al*, 1999; Salven *et al*, 1999b) and several reports have indicated, that elevated VEGF concentrations in serum may be a reflection of degranulation of platelets and white blood cells during *in vitro* clotting, rather than a reflection of an ongoing angiogenic activity in the tumour (Webb *et al*, 1998). In plasma, white cell and platelet degranulation is minimized by adding anticoagulatives to the blood samples, and as a consequence, plasma VEGF concentrations are up to 20 times lower than the matched serum VEGF concentrations (Banks *et al*, 1998). Therefore, it was suggested that plasma should be preferred as specimen for VEGF measurements and that serum was unsuitable (Banks *et al*, 1998).

The aims of the present study were to compare the prognostic significance of matched preoperative plasma and serum VEGF concentrations in patients with CRC and to evaluate whether serum or plasma was the best predictor of overall survival.

## MATERIALS AND METHODS

### Healthy volunteers

To establish the reference range among healthy people, matched plasma and serum VEGF concentrations were measured in 50 healthy volunteer blood donors. Their median age was 59 (55–65) years and there were 30 men and 20 women.

### Patients

The study included 524 consecutive patients scheduled to undergo elective resection of primary CRC. The median age of the patients at the time of operation was 69 (33–90) years, and 316 men and 208 women were included. All patients had their primary tumours resected and none were given chemotherapy or radiotherapy before or after the operation. All patients had histologically verified carcinoma localized in the colon or in the rectum and were staged according to the Dukes' classification with an added group D that identified patients with solid metastases or disease that had not been completely resected. The distribution of Dukes' stages according to site of tumour is shown in Table 1

**Table 1:**
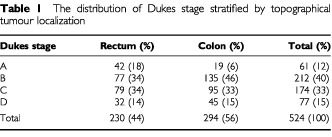
The distribution of Dukes stage stratified by topographical tumour localization

. The clinical data included overall survival for all patients, and because all Danes are given a computerized central personal registration number, none of the patients was lost to follow-up. The median follow-up time was 95 months (range 78–109) and 324 patients (62%) died during the observation period. The endpoint for survival analysis was death of all causes.

### Sampling of blood

The peripheral blood samples were collected in endotoxin-free silicone coated tubes (Becton-Dickinson, Mountain View, CA, USA) with EDTA as additive (plasma) or without additive (serum), after written informed consent in accordance with the Helsinki II declaration. The plasma samples were centrifuged (3000 r.p.m., 4°C, 10 min) immediately after the aspiration, and the plasma was removed and stored at −80°C until analyzed. The serum samples were allowed to clot at room temperature for 30 min before centrifugation (3000 r.p.m., 4°C, 10 min) and the serum was removed and stored at −80°C until analyzed. Blood samples were obtained preoperatively from the patients on the day of their operation, just before skin incision. The blood samples from the volunteer blood donors were obtained at the time of their routine donation of blood in the blood bank.

### VEGF analysis

Before analysis, the plasma and serum samples were thawed at room temperature. The VEGF concentration was measured with a commercially available human VEGF quantitative enzyme linked immunosorbent assay (ELISA) kit (R&D Systems, Minneapolis, MN, USA, Cat No: DVE00), according to the instructions given by the manufacturer. All analyses were made in duplicate and the mean value was used for statistical calculations.

### Statistical analysis

The SAS® software package (version 8.1; SAS Institute, Cary, NC, USA) was used to manage patient data and for statistical analysis. In the 524 CRC patients, the plasma and serum VEGF concentrations were scored as low if VEGF was less than or equal to the 95th percentile of the normal controls (112 and 533 pg ml^−1^ respectively) or otherwise scored as high. In addition, the patients were grouped into three strata by the 10th and 90th or the 25th and 75th percentiles of their plasma and serum VEGF concentrations. The end-point for survival analysis was death of any cause. The Kaplan–Meier method was used to estimate survival probabilities, and the log rank test was used to test for equality of strata. The Cox proportional hazard model was used for multivariate analysis. The assumption of proportional hazards was verified graphically. Rank statistics were used to calculate correlation coefficients and to test hypothesis on location. Tests of independence were done with the χ^2^ test. McNemars test was used for paired proportions. The level of significance was set at 5%.

## RESULTS

### VEGF concentrations in plasma

In the 50 healthy blood donors, the median plasma VEGF concentration was 30 pg ml^−1^ (range 0–369). There was no significant correlation (r_s_=0.009, *P*=0.95) between age and plasma VEGF among the blood donors and no significant difference in plasma VEGF (*P*=0.86) between men and women.

**Figure 1:**
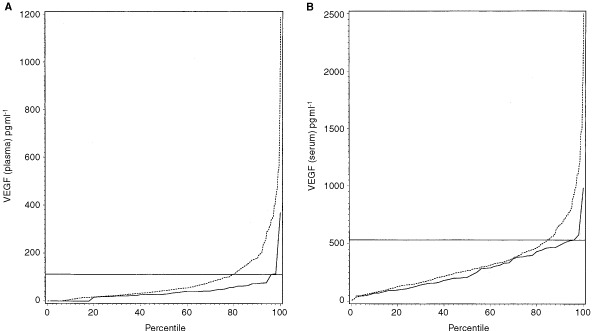
(**A**) Percentile plot of preoperative plasma VEGF levels in the 524 colorectal cancer patients and in the 50 healthy volunteer blood donors. The upper 95-percentile limit of healthy volunteer blood donors (continuous line) is added to the figure. (**B**) Percentile plot of preoperative serum VEGF levels in the 524 colorectal cancer patients and in the 50 healthy volunteer blood donors. The upper 95-percentile limit of healthy volunteer blood donors (continuous line) is added to the figure.

In the 524 patients with CRC, the median plasma VEGF concentration was 44 pg ml^−1^ (range 0–1185). There was no significant correlation (r_s_=0.05, *P*=0.21) between age and plasma VEGF among the CRC patients, and no significant difference in plasma VEGF (*P*=0.32) between men and women. The percentile plot of the plasma VEGF measurements in the 524 CRC patients and in the 50 blood donors is shown in Figure 1A

. The median preoperative concentration of plasma VEGF for patients with CRC was significantly (*P*=0.01) higher than the medium value for the healthy blood donors.

Stratified by Dukes stage, the median plasma VEGF concentration among the CRC patients was as follows: Stage A: 27 pg ml^−1^ (range 0–440); stage B: 44 pg ml^−1^ (range 0–1185); stage C: 44 pg ml^−1^ (range 0–706), and stage D: 74 pg ml^−1^ (range 0–543). The median plasma VEGF concentration significantly (*P*=0.001) increased with advanced Dukes stage. Patients with the primary tumour localized in the colon, had significantly (*P*=0.01) higher median plasma VEGF concentrations than patients with the primary tumour localized in the rectum.

### VEGF concentrations in serum

In the 50 healthy blood donors, the median serum VEGF concentration was 220 pg ml^−1^ (range 46–983). There was no significant correlation (r_s_=0.07, *P*=0.61) between age and serum VEGF among the healthy donors, and no significant difference in serum VEGF (*P*=0.74) between men and women was found.

In the 524 patients with CRC, the median concentration of serum VEGF was 268 pg ml^−1^ (range 9–2500). There was no significant correlation (r_s_=0.05, *P*=0.21) between age and serum VEGF among the patients, and no significant difference in serum VEGF (*P*=0.22) between men and women. The percentile plot of the serum VEGF measurements in the 524 CRC patients and in the 50 blood donors is shown in Figure 1B. Thus, the medium preoperative serum VEGF concentration in the CRC patients (268 pg ml^−1^) was higher than the medium serum VEGF concentration in the healthy controls (220 pg ml^−1^). However, in contrast to the median plasma VEGF concentration between CRC patients and healthy blood donors, the difference in the median serum VEGF concentration was not statistically significant (*P*=0.30).

Stratified by Dukes stage, the median serum VEGF concentration among the colorectal cancer patients was as follows: Stage A: 261 pg ml^−1^ (range 9–1500); stage B: 266 pg ml^−1^ (range 15–1975); stage C: 263 pg ml^−1^ (range 15–2500), and stage D: 304 pg ml^−1^ (range 19–1475). Patients with Dukes stage D disease had significantly (*P*=0.001) higher serum VEGF concentrations compared to patients with Dukes stage A, B and C disease, while the latter three groups had comparable concentrations. Patients with the primary tumour localized in the colon, had significantly (*P*=0.01) higher serum VEGF levels than patients with the primary tumour localized in the rectum.

### Correlation between matched preoperative serum and plasma VEGF concentrations

**Figure 2:**
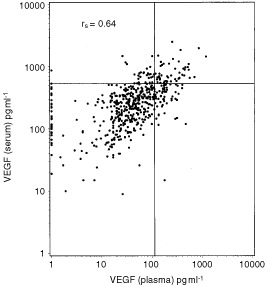
Scatter plot of the matched preoperative serum and plasma VEGF measurements in the 524 colorectal cancer patients. The rank correlation is shown (*P*<0.001) and plasma VEGF less than one are set to 1. In addition, the 95 percentile for plasma and serum VEGF of the healthy blood donors are shown.

The correlation (r_s_=0.64) between the 524 matched serum and plasma VEGF measurements is shown in Figure 2

. Dichotomizing VEGF in serum and plasma by the 95th percentile in the relevant control showed that the plasma measurements had significantly more positives than the serum measurements (*P*=0.01, McNemars test). This analysis indicated that, although serum and plasma concentrations are correlated, grouping by the 95th percentile of normal donors, significantly more positives are identified by plasma VEGF

### Prognostic significance of the preoperative VEGF level

**Figure 3:**
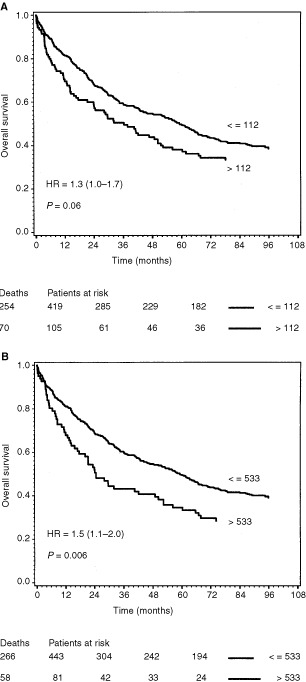
Survival curves of the 524 colorectal cancer patients dichotomized by the upper limit of the 95th percentile of healthy volunteer blood donors. The end-point for survival analysis was death of all causes. Differences between the two survival curves were assessed by the log rank test, the hazard rate with 95% confidence interval was calculated by the Cox regression model. The number of events in each group and the number of patients at risk after each 24-month interval up to 72 months is indicated below the curve. (**A**) The two curves represent patients with plasma VEGF values below or equal to 112 pg ml^−1^ (*n*=419, upper curve), and patients with plasma VEGF above this level (*n*=105, lower curve). (**B**) The two curves represent patients with serum VEGF values below or equal to 533 pg ml^−1^ (*n*=443, upper curve), and patients with serum VEGF above this level (*n*=81, lower curve).

**Figure 4:**
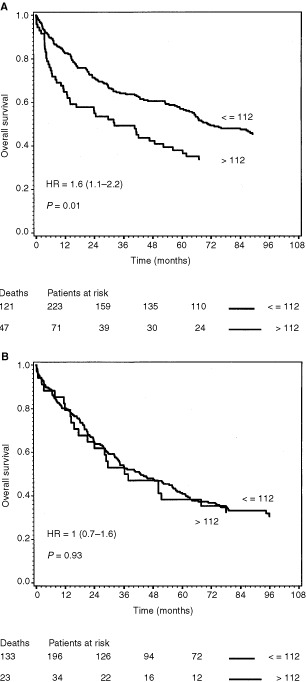
Survival curves of 294 colon cancer (**A**) and the 230 rectal cancer (**B**) patients dichotomized by the upper limit of the 95th percentile of healthy volunteer blood donors. The end-point for survival analysis was death of all causes. Differences between the two survival curves were assessed by the log rank test, the hazard rate with 95% confidence interval was calculated by the Cox regression model. The number of events in each group and the number of patients at risk after each 24-month interval up to 72 months is indicated below the curve. (**A**) The two curves represent colon cancer patients with plasma VEGF values below or equal to 112 pg ml^−1^ (*n*=223, upper curve), and colon cancer patients with plasma VEGF above this level (*n*=71, lower curve). (**B**) The two curves represent rectal cancer patients with plasma VEGF values below or equal to 112 pg ml^−1^ (*n*=196, upper curve), and rectal cancer patients with plasma VEGF above this level (*n*=34, lower curve).

**Figure 5:**
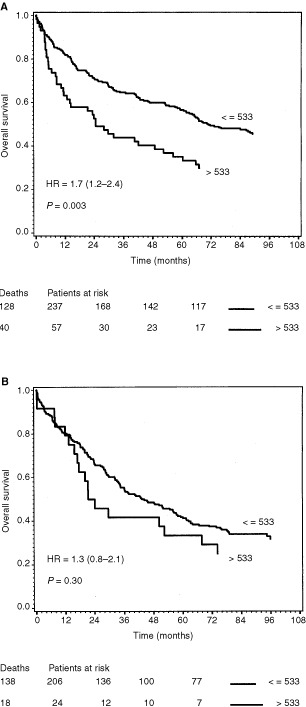
Survival curves of 294 colon cancer (**A**) and the 230 rectal cancer (**B**) patients dichotomized by the upper limit of the 95th percentile of healthy volunteer blood donors. The end-point for survival analysis was death of all causes. Differences between the two survival curves were assessed by the log rank test, the hazard rate with 95% confidence interval was calculated by the Cox regression model. The number of events in each group and the number of patients at risk after each 24-month interval up to 72 months is indicated below the curve. (**A**) The two curves represent colon cancer patients with serum VEGF values below or equal to 533 pg ml^−1^ (*n*=237, upper curve), and colon cancer patients with plasma VEGF above this level (*n*=57, lower curve). (**B**) The two curves represent rectal cancer patients with serum VEGF values below or equal to 533 pg ml^−1^ (*n*=206, upper curve), and rectal cancer patients with plasma VEGF above this level (*n*=24, lower curve).

In univariate analyses, by classifying the CRC patients in two groups, based on the upper VEGF limit of the 95th percentile of healthy controls, it was shown that patients with plasma VEGF concentrations above 112 pg ml^−1^ (*n*=105) had a reduced overall survival (although not significant, *P*=0.06) compared to the patients (*n*=419) with plasma VEGF concentrations equal to or below this level (Figure 3A

). In the group of patients with serum VEGF concentrations above the 95th percentile of healthy persons (533 pg ml^−1^), (*n*=81), overall survival was significantly (*P*=0.006) reduced, compared to patients with VEGF concentrations below or equal to this level (*n*=443) (Figure 3B). In the subgroup of patients with colon cancer, the patients with plasma VEGF above 112 pg ml^−1^ (*n*=71) had significantly (*P*=0.01) reduced overall survival, compared to colon cancer patients (*n*=223) with lower VEGF concentrations (Figure 4A

). This difference was not shown in patients with rectum cancer (*P*=0.93), (Figure 4B). Dichotomizing the serum VEGF, using the cut point 533 pg ml^−1^, the subgroup of patients with colon cancer and high VEGF concentration (*n*=57) had significantly (*P*=0.003) reduced survival compared to the patients (*n*=237) with low VEGF concentration (Figure 5A

). This difference was not observed in the subgroup of patients with rectal cancer (*P*=0.3) (Figure 5B).

**Figure 6:**
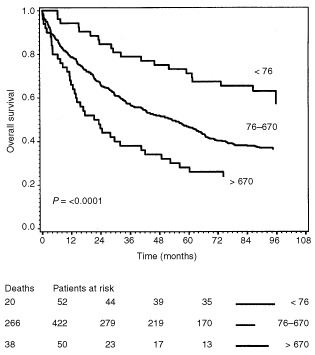
Survival curves of the 524 colorectal cancer patients grouped into three strata by the 10th and 90th percentiles of the preoperative serum VEGF concentrations. The end-point for survival analysis was death of all causes. Differences between the survival curves were assessed by the log rank test, the hazard rate with 95% confidence interval was calculated by the Cox regression model. The number of events in each group and the number of patients at risk after each 24-month interval up to 72 months is indicated below the curve. The three curves represent colorectal cancer patients with the following preoperative serum VEGF concentrations: I: <76 pg ml^−1^; II: 76−670 pg ml^−1^; III: >670 pg ml^−1^.

In the above-mentioned calculations, the cut-off point was set to the upper limit of the 95th percentile of healthy controls. However, this level may not be the relevant scoring. In order to study if a trend could be detected, strata were defined by the 10th and 90th percentiles of the plasma and serum VEGF concentrations. This division as seen in Figure 6

indicates that CRC patients with preoperative serum VEGF concentrations higher than 670 pg ml^−1^ had a significantly (*P*<0.0001) reduced survival compared to the patients with lower concentrations. Additionally, CRC patients with preoperative serum VEGF concentrations lower than 76 pg ml^−1^ had a significantly better prognosis than CRC patients with VEGF concentrations above this level. Using the 25th and the 75th percentiles for stratification shows a less pronounced effect although statistically significant (*P*=0.02). This effect was not seen with plasma VEGF.

### Multivariate analysis

Multivariate survival analysis was performed including Dukes stage, gender, age, topographical tumour localization, and VEGF level (Table 2

**Table 2:**
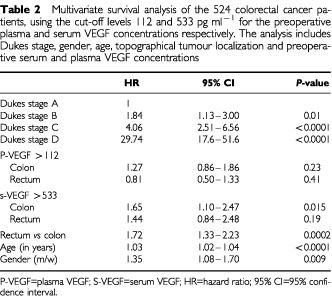
Multivariate survival analysis of the 524 colorectal cancer patients, using the cut-off levels 112 and 533 pg ml^−1^ for the preoperative plasma and serum VEGF concentrations respectively. The analysis includes Dukes stage, gender, age, topographical tumour localization and preoperative serum and plasma VEGF concentrations

). As expected, advanced Dukes stage was an independent predictor of overall survival. In patients with colon cancer, high preoperative serum VEGF (>533 pg ml^−1^) significantly (*P*=0.015) predicted a reduced overall survival, while high preoperative plasma VEGF level (>112 pg ml^−1^) did not (*P*=0.23). In patients with rectal cancer, neither high serum VEGF concentration nor high plasma VEGF concentration independently predicted a reduced overall survival (*P*=0.19 and 0.41 respectively). Additionally, patients with the tumour located in the rectum had a significantly (*P*<0.0001) worse prognosis than patients with the primary tumour located in the colon. In Table 3

**Table 3:**
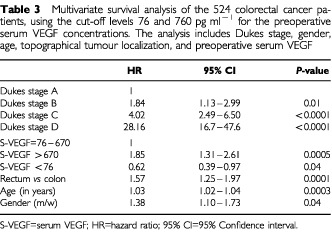
Multivariate survival analysis of the 524 colorectal cancer patients, using the cut-off levels 76 and 760 pg ml^−1^ for the preoperative serum VEGF concentrations. The analysis includes Dukes stage, gender, age, topographical tumour localization, and preoperative serum VEGF

, multivariate survival analyses using the cut-off points 76 and 670 pg ml^−1^ are shown. This survival analysis indicates that the 95th percentile may not be the relevant clinical cut-off point for serum VEGF. Furthermore, the analysis showed that the patients with the highest 10% of the serum VEGF concentrations had a significantly (*P*=0.0005) reduced survival compared to the CRC patients with lower VEGF concentrations, while the patients with the lowest 10% of the serum VEGF concentrations had a significantly (*P*=0.04) better prognosis than the patients with higher preoperative serum VEGF concentrations.

## DISCUSSION

The present study showed that preoperative concentrations of plasma and serum VEGF in patients with colorectal cancer were higher than in healthy controls, although the difference in the serum concentrations between the two groups was not significant. Additionally, the study indicated that high plasma and serum VEGF levels might be predictors of reduced overall survival in patients with CRC. In many aspects, the information obtained from the matched preoperative serum and plasma VEGF measurements were similar. The preoperative VEGF levels in plasma and serum were both higher than in healthy controls, and no correlation was found between gender and age in the two types of specimen. Additionally, in univariate survival analyses, high VEGF values predicted a reduced overall survival in both cases, although the preoperative serum concentration was a better predictor. Moreover, in the multivariate analyses, a high preoperative serum VEGF concentration, in the subgroup of patients with colon cancer, independently predicted a reduced overall survival, while high preoperative plasma VEGF concentration did not.

A significant (*P*<0.001, chi-square test) difference in Dukes stage was found between rectal and colon cancer patients, so that rectal cancer patients in general had a less advanced disease at the time of diagnosis. However, in an analysis including plasma and serum VEGF, topographical tumour localization and Dukes stage, patients with the tumour localized in the colon, independently of Dukes stage, still had significantly higher plasma and serum VEGF levels (*P*=0.001 and 0.0003 respectively, ANOVA) than patients with the tumour localized in the rectum. Therefore, the difference in plasma and serum VEGF levels between rectal and colon cancer is not simply a reflection of the difference in distribution of Dukes stage, but seems to be a genuine biological difference between the two tumour types.

Several studies have indicated that serum is unsuitable for VEGF measurements since the significantly higher VEGF concentration in serum, compared to matched plasma concentrations, may be due to release of VEGF from platelets during the *in vitro* coagulation (Banks *et al*, 1998; Webb *et al*, 1998). However, it has recently been shown that isolated platelets from cancer patients contain more VEGF than isolated platelets from healthy controls (Salven *et al*, 1999b) and a very recent study have demonstrated that the serum VEGF concentrations in cancer patients with normal platelet counts were higher than in healthy controls with normal platelet counts (Lee *et al*, 2000). Platelet aggregation may contribute to tumour progression by release of a variety of vasoactive and proangiogenic substances in the tumour (Browder *et al*, 2000) and to metastasis by facilitating adherence of disseminated tumour cells to capillary walls at distant sites (Hejna *et al*, 1999). Therefore, although the preoperative serum VEGF concentration is affected by *in vitro* degranulation of platelets, the increased platelet-derived VEGF may influence the biology of a present tumour *in vivo*, and may presumably reflect tumour burden at the time of surgery.

Previously, most clinical studies have addressed the prognostic impact of preoperative serum VEGF concentrations. Recently, it was demonstrated that plasma VEGF was increased in patients with colorectal cancer compared with controls (George *et al*, 2000) and that high plasma VEGF concentrations tended to occur with more advanced disease (Nakayama *et al*, 2000). The present study supports these observations and indicates furthermore, in a univariate analysis, that high concentrations may predict a reduced overall survival. However, in the multivariate analysis, in the subgroup of patients with colon cancer, high preoperative plasma VEGF concentration was not an independent predictor of reduced overall survival while a high preoperative serum VEGF concentration was. These findings may indicate that the preoperative serum VEGF concentration is a better prognostic parameter than the preoperative plasma VEGF concentration. However, since platelets and white cells in peripheral blood samples contribute to the concentration of VEGF in serum, a large prospective clinical study should investigate the prognostic significance of these parameters and their correlation to the VEGF concentration in serum and plasma.

## The following investigators participated in The RANXO5 Colorectal Cancer Study Group

Bispebjerg Hospital: S Schulze, MD, DMSc J Thorup, MD, P Wille-Jørgensen, MD, DMSc; Bornholm Hospital: E Bentzen, MD; Frederiksberg Hospital: I Christoffersen, MD, P Møller, MD; Frederikssund Hospital: L Banke, MD, DMSc, D Froberg, MD; Gentofte Hospital: FW Henriksen, MD, DMSc, P Crone, MD; Glostrup Hospital: P Hesselfeldt, MD, B Hempel Sparsø, MD, K Lindorff Larsen, MD; Helsingør Hospital: T Asmussen, MD, J Heiner, MD; Hillerød Hospital: O Hart Hansen, MD, DMSc, H Flyger, MD, PhD, P Jess, MD, DMSc; Holbæk Hospital: J Iversen, MD, J La Cour-Andersen, MD, DMSc, B Vennits, MD; Hvidovre Hospital: F Moesgaard, MD, DMSc, J Hammer, MD; Hørsholm Hospital: A Fischer, MD, DMSc, H Galatius, MD; Kalundborg Hospital: L Naver, MD; Køge Hospital: D Teilum, MD; Nykøbing Falster Hospital: L Holbraad, MD; Næstved Hospital: O Iversen, MD, DMSc, J Nymark, MD, O Roikjær, MD; Rigshospitalet: LB Svendsen, MD, DMSc, L Vedel, MD; Roskilde Hospital: L Palm, MD, DMSc, KC Rasmussen, MD; Slagelse Hospital: J Friis, MD, C Lanng, MD, K Wiboltt, MD; Stege Hospital: NC Jensen, MD, N Hoffman, MD; Sundby Hospital: T Larsen, MD, J Packler, MD.
